# Transitioning Traditions in the Time of COVID

**DOI:** 10.5811/westjem.2020.12.49118

**Published:** 2020-12-22

**Authors:** Michelle E. Romeo, Jeremy Branzetti

**Affiliations:** New York University, Bellevue Hospital, Department of Emergency Medicine, New York City, New York

In 1979 Drs. Lewis Goldfrank and Neal Lewin astutely perceived that the Bellevue Hospital emergency ward, the safety net of New York City, provided an exceptional learning environment for patient care. Every patient, in some capacity, was deemed an invaluable educational opportunity, and through this ideology the tradition of “Morning Report” was born. It would become a staple of the residency, and the department community as a whole, for the next 40 years.

Morning Report is a time-protected group learning experience led by a senior resident discussing a prior patient case. Learners in attendance are the students, residents, and attendings scheduled clinically for that day. Using the patient’s presentation, chief complaint, initial vital signs and physical exam, the resident strategically guides the learners through the case. Frequent pauses and active group engagement are used to generate a differential diagnosis, and seasoned providers discuss what their next steps would be given their knowledge of the details thus far. The tangential conversations that are sparked from the case allow trainees to understand the thought process that seasoned attendings use for patient care. The case usually ends with a discussion of the final diagnosis and best practices for us as providers. It is mostly during this time that learners sip on their coffee with wide eyes at the decades-old Bellevue conference table. It is also not uncommon to find attendings lingering behind to reminisce on prior cases or experiences that also left an impression on them or to learn a little bit more about the newest intern they meet that day. It was our opportunity to truly learn who our colleagues are that make up the community we are housed in.

Over the years, Morning Report has become so ingrained in the departmental culture that nursing and attendings provide clinical coverage during the early hour to protect time for learning. In the era of COVID-19, department volumes and acuity surged and our ability to take an hour away from shift was untenable. This, tied with the unwelcoming truth that our department could no longer gather in groups safely, led to a loss of an educational tool vital to our residency program and to our sense of community. With New York quickly becoming an epicenter of the early pandemic, web-based video conferencing became the new normal and the decision was made to trial Morning Report online. Using the platform of Zoom (Zoom Video Conferencing Inc., San Jose, CA), we recreated as much of the format as possible. Scheduling and clinical volumes necessitated moving away from morning learning: thus, a thrice-weekly “Evening Report” was born that would ultimately last eight weeks and carry us through dark, isolating nights of the pandemic.

While our cherished tradition of in-person learning was disrupted, we quickly realized the upside of remote connection: for the first time ever, those at affiliated sites, including advanced practice providers and nurses, were able to attend and learn alongside the residents. Alumni, who normally were just out of reach, returned “home” from all over the country to rebuild a new learning community with us. The new reality, while beneficial and relatively easy to implement, required adaptations. Residents, overwhelmed with the pandemic’s physical and emotional workload, were not expected to have much bandwidth to carry on another task. Instead of scheduling specific session leaders, a volunteer system was established. The expectation for slides and in-depth preparation was eschewed to promote on-the-spot learning that required minimal preparation. Volunteer leaders were only asked to bring a few details of the case to the table and allow the community to take over the discussion.

Our longtime facilitator, Dr. Lewin, was present at every evening report. This allowed at least one faculty member with decades of experience to help facilitate teaching. The sessions usually concluded with a comment from Dr. Goldfrank regarding best practices and summarizing our continued duties within this pandemic. Alumni eagerly stepped up to shoulder the teaching burden, asking to step in to fill in gaps, letting us know participation in this beloved tradition of their alma mater was not only wanted but savored. Inevitably, just like our in-person times, many hung around after the assigned presentation to check in with each other, socialize, and tell stories. Although slotted for an hour, the sessions would easily reach almost two hours. Alumni, attendings, and residents lingered to become more acquainted and reconnect with each other, recount stories of patient care, or simply share a virtual drink.

Participation was remarkable, with just over eight weeks at three sessions a week. Tradition was reborn. Anywhere from 20–50 participants attended each session. We learned that “Evening Report” was more than just a virtual adaptation of a long-standing tradition. Having a scheduled and protected educational event nightly brought back a sense of normalcy in a world that looked like nothing we had known. Being able to reconnect the department, affiliated sites, and alumni allowed the educational community not only to continue on but to flourish and grow.

Social isolation took a toll on us, with many having to be away from their families and friends. No gym class or department happy hour was available to heal the community or bring us together. So, while the initial intention of “Evening Report” was to carry on a tradition of those who have walked Bellevue’s halls for many years, it became a much more important venue for us to reconnect and support each other through a harrowing time of our medical careers. It became a time to come together to create true community wellness in a time of need. The “conference table” was back and allowed for more seats at the table than ever expected.

